# Pleomorphic Adenoma of Breast: A Radiological and Pathological Study of a Common Tumor in an Uncommon Location

**DOI:** 10.1155/2015/172750

**Published:** 2015-02-25

**Authors:** Paula S. Ginter, Theresa Scognamiglio, Pamela Tauchi-Nishi, Lilian B. Antonio, Syed A. Hoda

**Affiliations:** ^1^Department of Pathology and Laboratory Medicine, Weill Cornell Medical College, New York, NY 10065, USA; ^2^Department of Pathology, The Queen's Medical Center, Honolulu, HI 96813, USA

## Abstract

Pleomorphic adenoma occurs commonly in the major salivary glands but is uncommonly encountered in the breast. In both of these locations, the tumor is typically grossly circumscribed and has a “mixed” histological appearance, being composed of myoepithelial and epithelial components amid a myxochondroid matrix. Herein, we report a case of pleomorphic adenoma of the breast which was preoperatively thought to represent a fibroadenoma on clinical and radiological grounds. It is the rarity of the tumor in the breast, rather than its histological appearance, that causes diagnostic difficulty.

## 1. Introduction

Pleomorphic adenoma (PA) is a benign neoplasm which most commonly occurs in the parotid gland but has only rarely been described in the breast. PA is composed of two components, that is, myoepithelial and epithelial (hence, the synonym: mixed tumor). Both components are embedded in chondroid stroma [1]. The rarity of PA in the breast, as well as its unusual appearance, has contributed to misdiagnosis in this location [2, 3]. PAs are considered to be a variant of intraductal papilloma or adenomyoepithelioma [4] and are typically found in the subareolar region [5, 6]. PAs are generally indolent. Rare examples of malignant PA (i.e., carcinoma ex PA) in the breast have been reported [7].

## 2. Case Report

**Figure 1:**
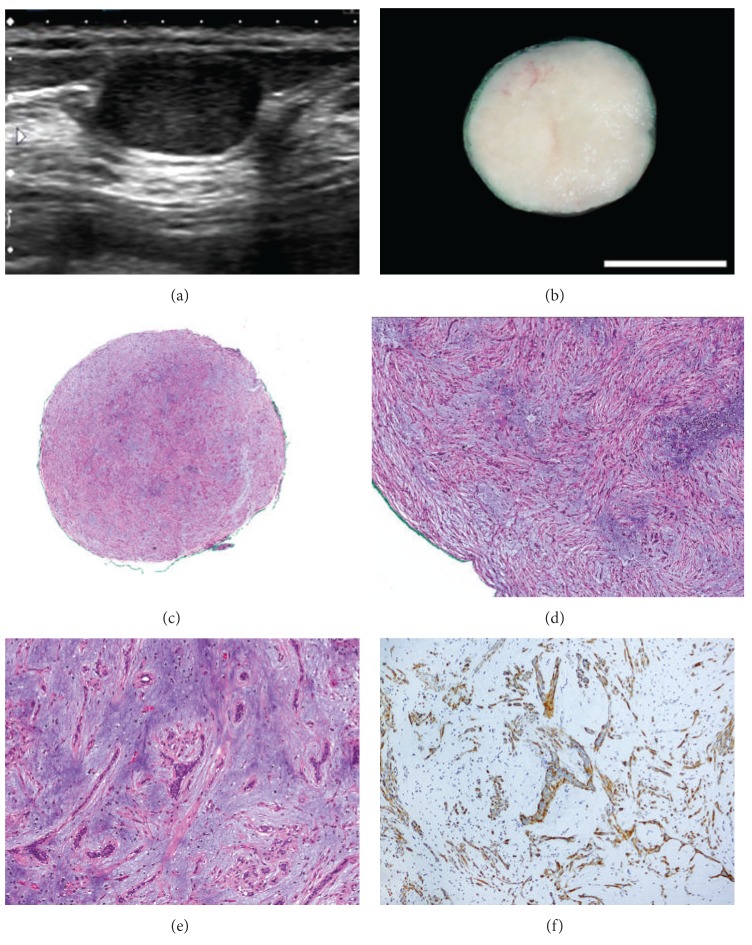
Pleomorphic adenoma of the breast. (a) Ultrasound showing a well-circumscribed, hypoechoic mass. (b) Cut sectioning showed a firm, well-circumscribed mass with whitish, homogeneous surface (bar: 1.0 cm). ((c) and (d)) Histological section shows the tumor to be composed of stromal and epithelial elements. The stromal component is comprised of bland spindle cells embedded in a myxochondroid matrix. (e) The epithelial component shows scattered compressed glands interspersed in the stroma. (f) Calponin immunostain highlights stromal and periglandular myoepithelial cells. The epithelial cells are negative for calponin.

A 42-year-old woman presented with a slowly growing palpable mass in the left breast. An ultrasound showed a well-circumscribed, hypoechoic mass that spanned 1.4 cm and was located at 1:00, 2 cm from the nipple (Figure 1(a)). The preoperative clinical and radiological impression was that of a fibroadenoma. A firm, well-circumscribed, 1.2 cm, mass was excised. Serial sectioning revealed whitish, homogeneous, cut surfaces (Figure 1(b), bar: 1.0 cm). Microscopically, the tumor was comprised of an admixture of stromal and epithelial elements. The dominant stromal component was characterized by bland spindled myoepithelial cells embedded in a largely myxoid, focally chondroid, matrix (Figures 1(c) and 1(d)). The epithelial component, represented by scattered compressed glands interspersed in the stroma, was cytologically insipid and mitotically quiescent (Figure 1(e)). The native breast glandular parenchyma, minimally represented in the specimen at the perimeter of the tumor, was inactive. There was no evidence of associated papilloma or adenomyoepithelioma. The lesional stromal myoepithelial cells and the periglandular myoepithelial cells were immunoreactive for calponin (Figure 1(f)), high molecular weight cytokeratin (CK-K903), and p63. The gross, histological and immunohistochemical findings in this tumor were characteristic of mammary PA. The most recent follow-up ultrasound examination of the ipsilateral breast, performed 5 months following the excisional biopsy, showed no evidence of residual or recurrent disease.

## 3. Discussion

PA is the most common benign tumor of the parotid gland; however, it is among the least common neoplasms of the mammary gland. In the latter location, it afflicts primarily adult females and typically presents as a solitary palpable central mass [3, 8, 9]. Published cases of mammary PAs have ranged in size from 0.6 cm to 17 cm, with most spanning ~2 cm [10–12]. PA can also occur, albeit most uncommonly, in the skin, vulva, and upper respiratory tract [13]. In all its primary locations, PA generally behaves in an indolent manner and neither recurs nor metastasizes following complete resection; nevertheless, at least 3 cases of malignant PA (i.e., carcinoma ex PA) in the breast have been reported [7]. In these cases of carcinoma ex PA, areas diagnostic of PA were present in addition to areas with histological features of malignancy as defined in the salivary gland counterpart [14]. The latter include infiltrative growth pattern, necrosis, marked cytological atypia, high mitotic rate, and presence of atypical mitoses [7]. It is notable that benign PAs of the breast have been mistaken for mucinous carcinoma [3] and metaplastic carcinoma [2], on limited samples of fine needle aspirates (FNA) and needle core biopsies (NCB), respectively—the perfidious myxoid matrix proving to be the diagnostic pitfall in these instances.

Although several sporadic clonal changes have been reported in PAs of the salivary gland, the most common chromosomal rearrangements therein involve 8q12, containing the target gene PLAG1, or 12q13-15 with the target gene HMGA2 [15]. The detection of PLAG1 and HMGAs translocations by either reverse transcriptase-polymerase chain reaction or fluorescent* in situ* hybridization can be useful in confirming the diagnosis in the rare diagnostically challenging PA in the salivary glands; however, the diagnostic utility of this technique at other sites remains uncertain.

In summary, we report a case of mammary PA—which was clinically and radiologically suspected to be a fibroadenoma. Histologically, the tumor demonstrated the characteristic histopathological features of a PA. Pathologists should keep this tumor in mind whenever a tumor with prominent myxochondroid appearance is encountered—particularly in aspiration cytology or needle core biopsy material.
